# Clinicopathologic and Immunohistochemical Findings from Autopsy of Patient with COVID-19, Japan

**DOI:** 10.3201/eid2609.201353

**Published:** 2020-09

**Authors:** Takuya Adachi, Ja-Mun Chong, Noriko Nakajima, Masahiro Sano, Jun Yamazaki, Ippei Miyamoto, Haruka Nishioka, Hidetaka Akita, Yuko Sato, Michiyo Kataoka, Harutaka Katano, Minoru Tobiume, Tsuyoshi Sekizuka, Kentaro Itokawa, Makoto Kuroda, Tadaki Suzuki

**Affiliations:** Toshima Hospital, Tokyo, Japan (T. Adachi, J.-M. Chong, M. Sano, J. Yamazaki, I. Miyamoto, H. Nishioka, H. Akita);; National Institute of Infectious Diseases, Tokyo, Japan (N. Nakajima, Y. Sato, M. Kataoka, H. Katano, M. Tobiume, T. Sekizuka, K. Itokawa, M. Kuroda, T. Suzuki)

**Keywords:** COVID-19, coronavirus disease, SARS-CoV-2, severe acute respiratory syndrome coronavirus 2, viruses, respiratory infections, zoonoses, cruise, autopsy, diffuse alveolar damage, immunohistochemistry, electron microscopy, next-generation sequencing, Japan

## Abstract

An autopsy of a patient in Japan with coronavirus disease indicated pneumonia lung pathology, manifested as diffuse alveolar damage. We detected severe acute respiratory syndrome coronavirus 2 antigen in alveolar epithelial cells and macrophages. Coronavirus disease is essentially a lower respiratory tract disease characterized by direct viral injury of alveolar epithelial cells.

Coronavirus disease (COVID-19), which was first reported in December 2019 in Wuhan, China, has been spreading rapidly and on a global scale. The causative virus is severe acute respiratory syndrome coronavirus 2 (SARS-CoV-2) (*1*). The World Health Organization declared the outbreak of COVID-19 to be pandemic on March 11, 2020, and had reported 693,282 laboratory-confirmed cases and 33,106 deaths globally as of March 30 (*2*). Numerous studies of the clinical features of COVID-19 and the virologic characteristics of SARS-CoV-2 have been conducted in China to date (*3*,*4*). Postmortem examination will provide valuable information required to elucidate the pathogenesis of COVID-19; however, only 2 studies have been published on COVID-19 pathology thus far (*5*,*6*). Further, the distribution of SARS-CoV-2 in a patient and identification of which cells are infected by SARS-CoV-2 have yet to be reported.

We describe the clinical course and the pathologic and virologic findings upon autopsy of a passenger on a cruise ship who died from COVID-19. The ship departed the port of Yokohama, Japan, on January 20, 2020, with a total of 3,711 passengers and crew; 712 (19%) of the persons on board were laboratory confirmed as having COVID-19. Of those, 12 had died as of March 31 (*7*).

## Case Report

**Figure 1:**
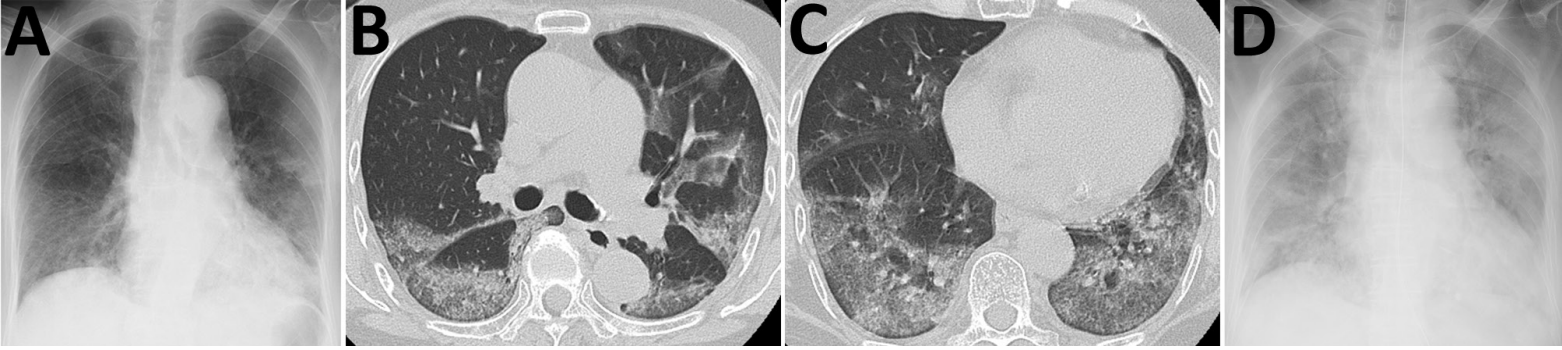
Chest radiograph and computed tomography results from an 84-year-old woman who died from coronavirus disease, Toshima Hospital, Tokyo, Japan, February 2020. A) Chest radiographs taken on admission (illness day 8), showing reticular shadows, mainly in bilateral lower lung fields. B, C) Chest computed tomography scan taken on illness day 8, indicating ground-glass opacities mainly located in posterior segments of the bilateral lower lobes, where the highest numbers of viral RNA copies were found on autopsy. D) Chest radiographs taken on illness day 14, with shadows spreading to almost entire lungs and exhibiting air bronchograms.

The passenger, an 84-year-old woman from Japan who had no notable medical history, had onset of fever (38.8°C) on February 5, followed by diarrhea (Table 1). On February 9, she went to the ship’s medical office with shortness of breath, and a throat swab sample was taken. Three days later (illness day 8), she was admitted to Toshima Hospital (Tokyo, Japan) with dyspnea on exertion; body temperature was 38.2°C, pulse rate 70 beats/min, blood pressure 156/80 mm Hg, respiratory rate 16 breaths/min, and oxygen saturation 95% (with 2 L/min oxygen supplementation). A chest radiograph showed opacities in both lungs, and a computed tomography scan revealed ground glass opacities and consolidations, mainly in bilateral lower lung lobes (Figure 1, panels A–C). The diagnosis of COVID-19 was confirmed by real-time reverse transcription PCR on the throat swab and reported on illness day 9. On illness day 10, hypoxia progressed, even with 15 L/min oxygen supplementation. The patient expressly stated that she did not want mechanical ventilation. Ampicillin/sulbactam was administered intravenously, based on the identification of *Klebsiella pneumoniae* and methicillin-sensitive *Staphylococcus aureus* by sputum culture. Corticosteroids were added after the appearance of progressive hypoxemia and acute respiratory distress syndrome. On illness day 13, the antiretroviral drug lopinavir/ritonavir was added orally. Despite all these treatment efforts, the dyspnea progressed and chest radiograph findings worsened (Figure 1, panel D). Intravenous morphine was initiated to alleviate breathing difficulties from illness on day 14. The patient died from respiratory failure on February 20 (illness day 16). The patient’s family gave consent for an autopsy to be performed.

**Table 1:** Symptoms, signs, laboratory results, and treatment administered for an 84-year-old woman who died from coronavirus disease, by day of illness, cruise ship and Toshima Hospital, Tokyo, Japan, February 2020*

Characteristic	Day of illness																
	Cruise ship								Hospital								
	1	2	3	4	5	6	7		8	9	10	11	12	13	14	15	16
Symptom																	
Temperature, °C	38.8				38.5		38.9		38.3	37.9	37.5	37.1	37.2	37.5	37.7	36.9	
Dyspnea†				+	+	+	+		++	++	++	++	+++	+++	+++	+++	+++
SaO_2_, %									95–96	90–95	86–94	84–92	83–86	79–84	74–84	66–79	
Intervention																	
O_2_ (L/min)									2	5	15	15	15	15	15	15	15
ABPC/SBT												*	*	*	*	*	
MPSL, HYD											*	*	*		*	*	*
LPV/r														*	*	*	
Morphine															*	*	*
Blood test result																	
Leukocytes, 10^3^/*µ*L									3.7		4.2			12.4			
PLT, 10^4^/*µ*L									13.4		14.0			22.8			
AST, U/L									53		58			46			
ALT, U/L									25		26			28			
CRE, mg/dL									0.71		0.64			0.63			
CRP, mg/dL									2.66		3.40			1.32			

*ABPC/SBT, ampicillin/sulbactam; ALT, alanine aminotransferase; AST, aspartate aminotransferase; CRE, creatinine; CRP, C-reactive protein; HYD, hydrocortisone; LPV/r, lopinavir/ritonavir; MPSL, methylprednisolone; PLT, platelet; SaO_2_, oxygen saturation. †Dyspnea severity indicated by +, mild; ++, moderate; +++, severe.

**Figure 2:**
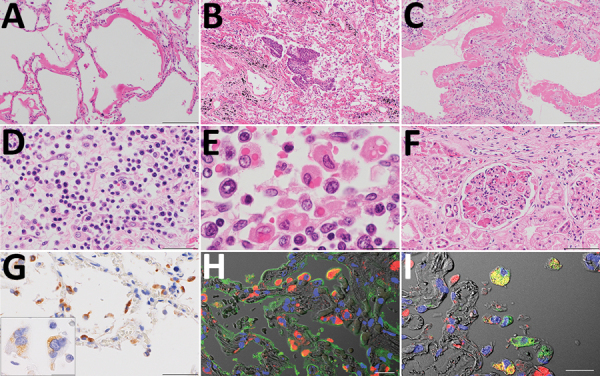
Pathologic findings for the lungs, lymph nodes, and kidneys in an autopsy of an 84-year-old woman who died from coronavirus disease, Toshima Hospital, Tokyo, Japan, February 2020. A) Marked diffuse alveolar damage in exudative phase with prominent hyaline membrane formation in lung tissues. Hematoxylin & eosin (H&E) staining. Scale bar indicates 200 µm. B, C) Desquamation and squamous metaplasia of the epithelium (B) and organized hyaline membranes (C), with septal fibrosis in the organizing phase lesions in lung sections. H&E staining. Scale bar indicates 200 µm. D) Inflammatory infiltrate comprised predominately of plasma cells in the alveolar septa. H&E staining. Scale bar indicates 50 µm. E) Obvious erythrophagocytic macrophages in the lymph nodes. H&E staining. Scale bar indicates 20 µm. F) Numerous microthrombi in the glomerulus in the kidneys. H&E staining. Scale bar indicates 100 µm. G) Immunostaining (brown) of severe acute respiratory syndrome coronavirus 2 antigen in alveolar epithelial cells. Scale bar indicates 50 µm. Inset: multinucleated syncytial cells; scale bar indicates 20 µm. H, I) Double immunofluorescence staining for severe acute respiratory syndrome coronavirus 2 (red) with epithelial cell marker (H; epithelial membrane antigen staining, green); macrophage marker (I; anti-CD68 antibody staining, green) in the same cell. TO-PRO-3 nucleic acid staining (blue) and differential contrast images are also shown. Scale bar indicates 20 µm.

An autopsy was conducted 5 hours after death, with the exception of the brain and bone marrow. Macroscopically, the trachea and bronchi exhibited neither redness nor erosion; however, the lungs (left, 590 g; right, 690 g) were partially dark red, consolidated, and airless. The cut surface was slightly sticky. Specifically, both pleurae were slightly thickened, with pleural effusions of <1 mL in each pleural cavity. The heart (420 g) showed right ventricular dilatation, with 10 mL of cardiac effusion. We noted diffuse multiple punctate hemorrhages in the mucosa of the stomach and duodenum. Histologic analysis revealed that the lungs exhibited features of both exudative and organizing diffuse alveolar damage (DAD). The lung tissues in the exudative phase of DAD showed prominent hyaline membranes (Figure 2, panel A), and those in the organizing phase of DAD showed desquamation, squamous metaplasia of the epithelial cells (Figure 2, panel B), organizing hyaline membranes (Figure 2, panel C), and inflammatory cell infiltration with prominent plasma cells in the alveolar septa (Figure 2, panel D). We observed intra-alveolar hemorrhage, vascular congestion, and hyperplasia of type 2 pneumocytes. We also noted multinucleated syncytial cells. In addition, we detected hemophagocytosis in the lungs, spleen, and lymph nodes (Figure 2, panel E). The glomeruli of both kidneys were marked by microthrombi, suggesting early signs of disseminated intravascular coagulation (Figure 2, panel F). We observed no notable changes in the other organs.

To examine the distribution of SARS-CoV-2 antigens, we performed immunohistochemistry on all tissue sections by using rabbit polyclonal antibodies against the SARS-CoV nucleocapsid protein (*8*). We confirmed the reactivity of the antibody by using SARS-CoV-2–infected VeroE6/TMPRSS2 cells as a positive control and a mock-infected VeroE6/TMPRSS2 cells as a negative control (*9*). We detected SARS-CoV-2 antigens in the cytoplasm of alveolar epithelial cells in earlier-stage DAD lesions, with mild inflammation before formation of hyaline membranes (Figure 2, panel G) rather than progressed lesions. We also detected viral antigens detected in the cytoplasm of multinucleated syncytial cells (Figure 2, panel G, inset). We detected no signals in the trachea, intestine, or other extrapulmonary tissue sections. Double immunofluorescence staining revealed that the viral antigen was present in epithelial membrane antigen-positive alveolar epithelial cells and CD68 (clone PGM-1)–positive alveolar macrophages (Figure 2, panels H and I).

We determined copy numbers of SARS-CoV-2 RNA in various specimens by using real-time reverse transcription PCR to amplify a segment in the nucleocapsid protein–encoding region of SARS-CoV-2 RNA, using forward (5′-GGCCGCAAATTGCACAAT-3′) and reverse (5′-CCAATGCGCGACATTCC-3′) primers, and a labeled probe 5′-(FAM)-CCCCCAGCGCTTCAGCGTTCT-(TAMRA)-3′ (Table 2). We collected postmortem tissues by using a new set of forceps and scissors for each sample to avoid cross-contamination. We used the amount of human glyceraldehyde-3-phosphate dehydrogenase mRNA in the RNA extracted from each tissue as an internal reference for normalization. Although SARS-CoV-2 RNA loads in serum samples increased from illness day 8 to day 13, at the time of autopsy, we detected SARS-CoV-2 RNA at low levels in whole blood and feces but not in urine. The copy numbers of SARS-CoV-2 RNA detected in the swab samples collected during the autopsy were higher in the right bronchus than in the nasopharynx. In addition, SARS-CoV-2 RNA–glyceraldehyde-3-phosphate dehydrogenase mRNA ratios in each tissue sample showed that viral loads in peripheral lung tissues were higher than those in trachea, bronchi, and upper respiratory tract tissues. We also detected low levels of SARS-CoV-2 RNA in nonrespiratory tract tissues, including the colon, liver, and spleen. Whole-genome sequencing of SARS-CoV-2 from the lung of the patient did not indicate substantial mutations except for a few single-nucleotide variations, including G11083T transversion compared with Wuhan-Hu-1 (GenBank accession no. MN908947; GISAID identification no. EPIISL402125), which is shared by the isolates obtained from the Diamond Princess cruise ship outbreak.

**Table 2:** Quantification of SARS-CoV-2 RNA in multiple specimens from an 84-year-old woman who died from coronavirus disease, Toshima Hospital, Tokyo, Japan, February 2020*

Day of illness	Specimen type or site	SARS-CoV-2 RNA	SARS-CoV-2, copies/reaction	GAPDH, copies/reaction	SARS-CoV-2 to GAPDH, ratio
Day 8 (admission)	Serum, copies/μL	2.7 × 10^1^			
Day 10		6.2 × 10^1^			
Day 13		2.8 × 10^2^			
Day 16 (autopsy)	Whole blood, copies/μL	1.6 × 10^2^			
	Urine, copies/μL	UDL			
	Feces, copies/μL	1.2 × 10^2^			
	Swabs, copies in 1 μL medium)				
	Nasopharynx	2.9 × 10^3^			
	Trachea	1.5 × 10^2^			
	Right bronchus	6.6 × 10^4^			
	Left bronchus	1.3 × 10^2^			
	Rectum	3.7 × 10^1^			
	Frozen tissues				
	Pharynx		83	2,670	3.1 × 10^–2^
	Tonsils		UDL	3,730	NA
	Epiglottis		43	13,000	3.3 × 10^–3^
	Trachea		UDL	145	NA
	Right bronchus		840	421	2.0 × 10^0^
	Right lung				
	Upper, S1/S2		76,600	1,570	4.9 × 10^1^
	Upper, S3		584	82	7.1 × 10^0^
	Middle, S5		11,900	3,000	4.0 × 10^0^
	Lower, S6		37,100	1,230	3.0 × 10^1^
	Lower, S8/S9		21,500	1,860	1.2 × 10^1^
	Lower, S7/S10		17,100	221	7.7 × 10^1^
	Left bronchus		67	93	7.2 × 10^–1^
	Left lung				
	Upper, S1+2		56,500	2,300	2.5 × 10^1^
	Upper, S3		26,300	12,600	2.1 × 10^0^
	Upper, S4/S5		6,260	1,530	4.1 × 10^0^
	Lower, S6		80,500	1,840	4.4 × 10^1^
	Lower, S8/S9		1,000	325	3.1 × 10^0^
	Lower, S10		22,900	977	2.3 × 10^1^
	Heart		UDL	64,800	NA
	Liver		57	104,000	5.5 × 10^–4^
	Kidney		UDL	5,910	NA
	Spleen		259	444,000	6.0 × 10^–4^
	Pancreas		UDL	3,690	NA
	Colon		35	5, 030	7.0 × 10^–3^

*GAPDH, glyceraldehyde-3-phosphate dehydrogenase; NA, not applicable; S, segment; SARS-CoV-2; severe acute respiratory syndrome coronavirus 2; UDL, under detection limit (<10 copies/reaction)**.**

## Conclusions

We report an autopsy of an 84-year-old cruise ship passenger who died from COVID-19. Lung pathology showed exudative and organizing phases of DAD, similar to what is observed in cases of severe acute respiratory syndrome (*10*–*13*). We detected SARS-CoV-2 antigen in alveolar epithelial cells and alveolar macrophages, also similar to what is observed in cases of severe acute respiratory syndrome (*14*,*15*). COVID-19 begins with upper respiratory tract symptoms (*3*) and ultimately becomes a lower respiratory tract disease in the later stages, based on the higher copy numbers of SARS-CoV-2 in the lower respiratory tract, relative to serum, whole blood, urine, feces, and rectal swab specimens taken during the clinical course and after death. COVID-19 is probably caused by direct injury of alveolar epithelial cells by SARS-CoV-2, accompanied by secondary damage to nonrespiratory organs. The high prevalence of SARS-CoV-2 infection on the cruise ship could not be attributed to specific genetic mutations of the virus.
